# Mid-infrared-scanning cavity ring-down CH_2_F_2_ detection using electronically tuned Cr:ZnSe laser

**DOI:** 10.1038/s41598-022-12019-5

**Published:** 2022-05-12

**Authors:** Masaki Yumoto, Yasushi Kawata, Satoshi Wada

**Affiliations:** 1 Photonics Control Technology Team, RIKEN Center for Advanced Photonics, RIKEN 2-1 Hirosawa, Wako, Saitama 351-0198 Japan; 2 Mid-Infrared Laser Source Laboratory, RIKEN Baton Zone Program, RIKEN, 2-1 Hirosawa, Wako, Saitama 351-0198 Japan

**Keywords:** Environmental monitoring, Infrared spectroscopy, Mid-infrared photonics, Optical sensors

## Abstract

The development of mid-infrared (mid-IR) tunable lasers has been driving various laser spectroscopic technologies. Herein, we report wavelength-scanning cavity ring-down spectroscopy (WS-CRDS) in the mid-IR region using an electronically tuned Cr:ZnSe (ET-Cr:ZnSe) laser, which could achieve a nanosecond pulse operation, with broad wavelength tuning of 2–3 µm. This allowed WS-CRDS-induced trace detection of the refrigerant, CH_2_F_2_. A CH_2_F_2_ detection limit of 0.66 ppm (3σ), and the detection of trace H_2_O in CH_2_F_2_ was realized using the broad wavelength-tuning range feature, demonstrating the effectiveness of the ET-Cr:ZnSe laser in WS-CRDS. We believe that our method would accelerate the development of various trace-gas detection technologies.

## Introduction

With the popularization of air-conditioning systems, refrigerants have become indispensable. Currently, hydrofluorocarbons (HFCs), which are chlorofluorocarbons (CFCs) and hydrochlorofluorocarbons (HCFCs) replacements^1,2^ and exhibit a low ozone depletion potential (ODP), are mainly used as refrigerants. However, HFCs are subjected to reduction and emission control regulations under the Montreal and Kyoto Protocol because of their flammability and high global warming potential (GWP)^3,4^. Their leakage monitoring, crucial for environmental conservation and climate research, necessitates the development of trace refrigerant detection technology.

Although cavity ring-down spectroscopy (CRDS)^5,6^, photoacoustic spectroscopy^7^, and tunable diode laser absorption spectroscopy with multipass gas cells^8^ are employed for high-sensitive detection of various trace volatile organic compounds (VOCs), their implementation in trace HFC detection has not yet been demonstrated. Among these methods, CRDS is a widely used optical sensing technique for ultrahigh-sensitivity measurements, wherein the effective optical path length can be significantly increased using a highly reflective cavity. To detect trace HFCs using CRDS, a laser that can tune the wavelength to the mid-infrared (IR) absorption peak of HFCs is required. Some HFCs exhibit strong absorptions in the mid-IR region (wavelength > 3 µm)^9–11^. Recently, strong absorptions at 2.1–2.5 µm have been noticed for difluoromethane (CH_2_F_2_), 2,3,3,3-tetrafluoroprop-1-ene (CH_2_=CFCH_3_), and (E)-1,3,3,3-tetrafluoroprop-1-ene (CHF=CHCF_3_)^12^. CH_2_=CFCH_3_ and CHF=CHCF_3_ are hydrofluoroolefins (HFOs) that are new candidates for refrigerants. Compared to low-molecular-weight gases (e.g., CH_4_, C_2_H_4_, and CO_2_), HFCs exhibit broad absorption spectra under ordinary temperature and pressure. In CRDS, distributed feedback lasers operate at a single frequency, and quantum cascade lasers are used for detecting low-molecular-weight gases^13,14^. However, the wavelength tuning ranges (e. g., 1–5 nm) of these lasers are narrower than the absorption spectral width of the HFCs; thus, the entire absorption spectrum of HFCs, spanning several 10-nm spectral regions, cannot be measured and the identification of the impurities based on the absorption spectral shape becomes difficult. For trace HFC detection using CRDS, a laser with a broad wavelength tuning range that includes the entire absorption spectral region is effective. However, increasing the CRD mirror reflectance to obtain a high sensitivity drastically reduces the CRDS signal intensity. Therefore, high-intensity and broadly tunable mid-IR lasers are required for trace HFC.

Cr^2+^-doped chalcogenides (e.g., Cr:ZnSe, Cr:ZnS, and Cr:CdSe), enabling direct access to the spectral range of 2–3 μm because of their broad fluorescence region and large stimulated-emission cross sections, have been used as the laser media in mid-IR tunable lasers^15,16^. These media prompted the development of high-power tunable lasers and ultrashort pulse lasers in the mid-IR region, without using nonlinear frequency conversion techniques^17–19^. Among these lasers, the 2–3 µm tunable nanosecond pulsed lasers are highly adaptable for the trace detection of CH_2_F_2_ using CRDS. Furthermore, because the output power per unit wavelength is higher than that of the supercontinuum laser, CRDS with a high signal-to-noise ratio can be realized. An electronically-tuned Cr:ZnSe (ET-Cr:ZnSe) laser equipped with an acousto-optic tunable filter (AOTF) as a wavelength tuning element reportedly produced a tuning in the range 2.17–2.71 µm and pulse energy of 7.9 mJ at 2.41 µm, which demonstrates a rapid wavelength tuning using a computer program^20^. Using Cr:CdSe, a grating-tuned laser with a broad tuning range of 2.25–3.08 μm and output energy of 4 mJ at 2.64 μm has been demonstrated^21^. However, these lasers are unsuitable for CRDS because the relaxation oscillations modulate the temporal profiles of the nanosecond pulses into multipeak pulses. Mirov et al.^22^ reported a broadly tunable Cr:ZnS laser using a Q-switched Er:YAG laser with a 50 ns pulse width, showing an output energy of 7 mJ at 2.25 μm and a tuning range of 1.95–2.65 μm via grating rotation. Although it can be applied to CRDS, the grating tuning method is inferior in wavelength controllability to the electronic tuning method in terms of wavelength-tuning speed, wavelength reproducibility, and random-wavelength switching. Both optimal temporal profile and high wavelength controllability are required for wavelength-scanning CRDS (WS-CRDS) in the 2–3 µm region.

Herein, we report trace CH_2_F_2_ detection using CRDS with an ET-Cr:ZnSe laser, which was improved to perform single-peak nanosecond pulse operation. We also demonstrate the detection of CH_2_F_2_ and H_2_O via the rapid wavelength scanning of the ET-Cr:ZnSe laser. Thus, the ET-Cr:ZnSe laser is an effective light source for WS-CRDS in the mid-IR region.

## Results and discussion

### WS-CRDS system using ET-Cr:ZnSe laser

**Figure 1:**
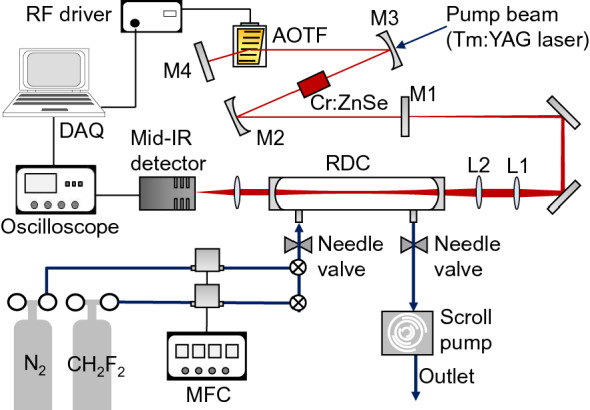
Schematic of the experimental setup. *AOTF* acousto-optic tunable filter, *M1* output coupler, *M2 and M3* folding mirrors, *M4* total reflector, *L1 and L2* lens pair, *RDC* ring-down cavity, *MFC* mass flow controller. The footprint of the ET-Cr:ZnSe laser and CRDS setup was 90 cm × 60 cm.

A schematic of the WS-CRDS setup for CH_2_F_2_ detection, composed of an ET-Cr:ZnSe laser, a ring-down cavity (RDC), a mid-IR detector (PVI-4TE-4, VIGO system), and a gas supply system, is shown in Fig. 1. We used a 15 mm-long antireflection-coated (for 1.5–2.7 µm) polycrystalline Cr:ZnSe (IPG Photonics, Inc.) as the laser medium with a Cr^2+^ doping concentration of 9.0 × 10^18^ cm^−3^. A Z-fold laser cavity configuration was developed using Cr:ZnSe, two folding mirrors (Concave radius = 500 mm), a total reflector, an output coupler, and an AOTF (Gooch & Housego). The output coupler and total reflector were flat, showing 70 and 99.5% reflections, respectively, for 2.1–3.5 μm. The folding mirrors were high-reflection-coated for 2.1–3.5 μm. We used a laser-diode-pumped 2 µm Q-switched Tm:YAG laser with a 10 Hz repetition rate as the pump source^23^, exhibiting maximum pulse energy of 1.8 mJ with a 53 ns pulse duration. To tune the wavelength, the AOTF was placed inside the cavity and connected to a radiofrequency (RF) driver whose power could be adjusted using a scanning computer program. This AOTF-based electronic tuning method promises high-speed continuous- or random-access tuning over a broad mid-IR region^20^. The laser output was passed through a lens pair and input to a ring-down cavity (RDC) consisting of two highly reflective mirrors placed 50 cm apart, each with a concave radius of 1000 m and a specified reflection of > 99.99% at 2.64 µm (LohnStar Optics, Inc.). The output beam from the RDC was focused on the mid-IR detector. The inlet and outlet of the RDC were connected to the gas supply and scroll pumps, respectively. Among the HFCs, we selected CH_2_F_2_ as the sample gas because it is mainly used in air-conditioning systems. CH_2_F_2_ exhibits a zero ODP and a GWP of 677, which is approximately 1/3rd of the GWP of CFCs and HCFCs^24^. The gas supply system was composed of a mass flow controller and reference gases (N_2_ and CH_2_F_2_). The CH_2_F_2_ concentrations were adjusted by controlling the flow rate of each reference gas. The ring-down signals were acquired on a computer using LabVIEW. All the experiments were performed at atmospheric pressure.

### Output performances of ET-Cr:ZnSe laesr

**Figure 2:**
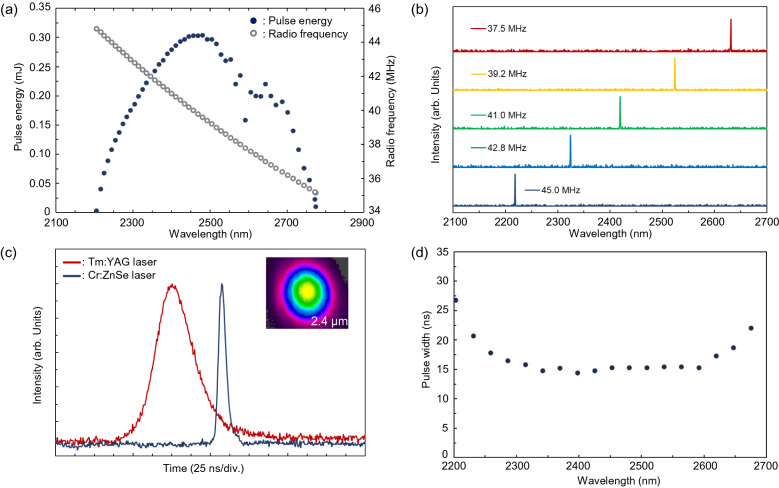
Output performances of the ET-Cr:ZnSe laser. (**a**) Wavelength tuning range and filter tuning curve of the AOTF. (**b**) Output optical spectra of the ET-Cr:ZnSe laser. (**c**) Temporal and spatial profiles at 2.4 µm. (**d**) Pulse width as a function of wavelength.

Figure 2 shows the output performance of the ET-Cr:ZnSe laser. The wavelength-tuning range and the filter tuning curve of ET-Cr:ZnSe laser are shown in Fig. 2a. We measured the wavelength and output energy by changing the RF signals fed into the AOTF. The wavelength was measured using a wavemeter (IR-III WS6-200, HighFinesse). The wavelength tuning range of 2.21–2.77 μm was achieved by changing the RF signals between 35.2 and 44.8 MHz. The maximum output energy of 0.3 mJ was obtained at 2.41 µm under a 1.8-mJ pumping. The decline of the output energy at approximately 2.6 µm is caused by water vapor absorption in the atmosphere. Figure 2b shows the output optical spectra of the ET-Cr:ZnSe laser. For the RF signals of 37.5, 39.2, 41.0, 42.9, and 45.0 MHz, the spectral peaks at 2631.3, 2523.9, 2419.1, 2324.3, and 2218.5 nm were measured, respectively, using a spectrometer (ASP-IR-3.5, AVESTA Ltd.), and the spectral width at each wavelength was 1–1.5 cm^−1^. The temporal and spatial profiles at 2.4 µm are shown in Fig. 2c. The ET-Cr:ZnSe laser drove 14 ns pulses at 2.4 µm under 53 ns pumping pulses, and the spatial beam profile in the TEM_00_ mode was observed. In the ET-Cr:ZnSe laser reported in^20^, the relaxation oscillations modulate the temporal profiles of the nanosecond pulses into multipeak pulses due to the long pump duration (${\tau }_{p}$= 300 ns). However, in this study, the short pump duration (${\tau }_{p}$= 53 ns) suppresses the relaxation oscillations and provides single peak pulses. Figure 2d shows the pulse duration as a function of wavelength. Pulse durations of 14–20 ns were observed in 2250–2650 nm, whereas those longer than 20 ns were observed in the outer regions of 2250 and 2650 nm owing to the small stimulate-emission cross-sections of Cr:ZnSe.

### WS-CRDS CH_2_F_2_ detection

**Figure 3:**
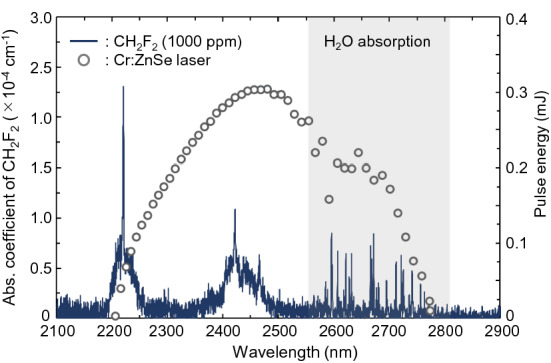
CH_2_F_2_ absorption spectrum measured by FTIR spectroscopy. The gray plots show the wavelength tuning range of the ET-Cr:ZnSe laser. The absorption spectrum of H_2_O was also observed in 2550–2800 nm. The gas pressure inside the gas cell was set to 1 atm.

The absorption spectrum of the CH_2_F_2_ (1000 ppm) reference gas is shown in Fig. 3. A Fourier transform infrared (FTIR) spectrometer equipped with a multipass gas cell (optical pass length = 12 m) was used to measure the absorption spectrum. The absorption peaks of CH_2_F_2_ appear at approximately 2220 and 2422 nm. The absorption spectra observed in the 2600–2800 nm region originated from the H_2_O impurity. The absorption peak at 2422 nm is around the gain center of the ET-Cr:ZnSe laser. The CRDS-based detection of CH_2_F_2_ was performed at 2422 nm to obtain high sensitivity and signal-to-noise ratios.

**Figure 4:**
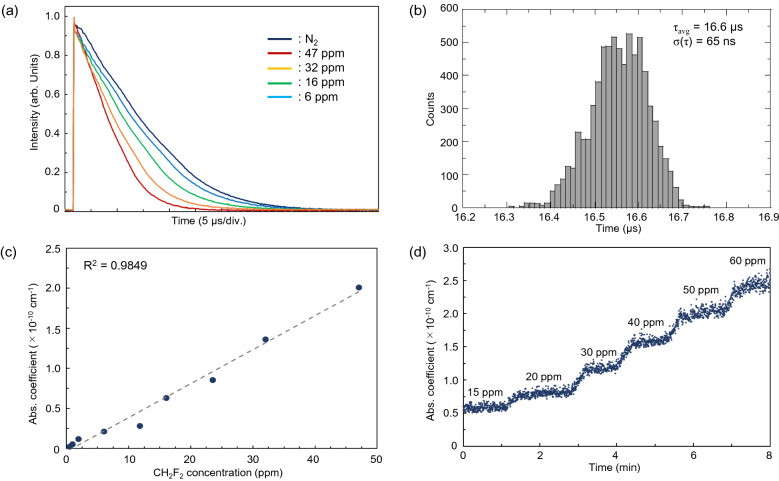
CH_2_F_2_ detection using CRDS with the ET-Cr:ZnSe laser. (**a**) RDTs under changing CH_2_F_2_ concentrations. Each RDT was obtained by averaging 32 ring-down events. (**b**) Histogram of the RDTs in the N_2_-purged RDC. In total, 7300 ring-down events were used. (**c**) Linear regression plot between the CH_2_F_2_ concentration and the absorption coefficient. Each RDT was obtained by averaging 32 ring-down events. (**d**) Continuous measurement results with cascading CH_2_F_2_ concentration changes.

The ring-down times (RDTs) at which CH_2_F_2_ and N_2_ (99.999%) were introduced into the RDC are shown in Fig. 4a. An RDT of 16.6 µs was measured when the RDC was purged with N_2_. As the CH_2_F_2_ concentration inside the RDC increased from 6 to 47 ppm, the RDTs decreased from 14.4 to 8.3 µs. From the experimentally obtained RDT, the effective reflectivity and optical length of the RDC were estimated to be R = 99.99% and *l*_*eff*_ = 5 km, respectively. This reflectivity is reasonable compared to the specifications provided by the vendor, according to the relationship $\tau =l/c(1-R)$, where *l* is the RDC length. Figure 4b shows a histogram of the RDTs in the N_2_-purged RDC at 2422 nm. The average and standard deviation ($\sigma (\tau )$) of the RDT were 16.6 µs and 65 ns, respectively. The noise-equivalent absorption sensitivity (NEAS)^25^ of the CH_2_F_2_ detection was calculated as $\mathrm{NEAS}= \sigma (\tau )/c\cdot {\tau }^{-2}\cdot {{f}_{a}}^{-1/2}$, where $\sigma (\tau )$ is the standard deviation of the RDT ($\tau$) in the N_2_-purged RDC, $c$ is the speed of light, and ${f}_{a}$ is the RDT acquisition rate; $\sigma (\tau )/\tau$ = 0.39% was obtained from the histogram, indicating NEAS ≈ 2.5 × 10^–9^ cm^-1^ Hz^-1/2^ (${f}_{a}=10 \,\mathrm{Hz}$). Figure 4c shows a linear regression plot of the CH_2_F_2_ concentration and the absorption coefficient. The CH_2_F_2_ concentrations were adjusted in the range of 0.5–47 ppm by changing the mixing ratio of the CH_2_F_2_ (100 ppm) and N_2_ (99.999%) reference gases using a gas dilution system. A linear fit to the experimental data yielded an R^2^ value of 0.9849, indicating an excellent linear response of the sensor. We estimated the detection limit within three standard deviations (3σ)^26^. The 3σ value of ~ 0.2 µs, obtained from the histogram, corresponded to a concentration of 0.66 ppm when compared with the linear-fitting line shown in Fig. 4c. Figure 4d shows the results of the continuous measurement of CH_2_F_2_ absorption when its concentration was changed gradually. The total flow rate of CH_2_F_2_ (100 ppm) and N_2_ (99.999%), provided by the gas supply system, was kept constant at 1 L/min, and the mixing ratio of CH_2_F_2_ and N_2_ was gradually changed from 0.15 L/min:0.85 L/min to 0.6 L/min:0.4 L/min. We succeeded in monitoring the continuous changes in the CH_2_F_2_ concentration in the RDC, implying that it is possible to monitor the CH_2_F_2_ leakage constantly for a ppm-level concentration.

**Figure 5:**
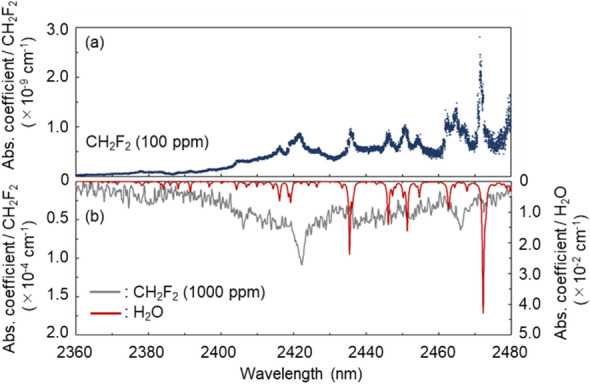
(**a**) CRD absorption spectrum of CH_2_F_2_ (100 ppm) in the 2360–2480 nm region. (**b**) CH_2_F_2_ (1000 ppm) absorption spectrum measured by FTIR spectroscopy (gray curve) and pure H_2_O absorption spectrum simulated using the HITRAN database (red curve).

The CRD absorption spectrum of CH_2_F_2_ (Fig. 5a) was obtained by measuring the RDTs while scanning the ET-Cr:ZnSe laser wavelength in 2360–2480 nm, which was realized by sweeping the RF signals (39.62–41.76 MHz) fed into the AOTF. The scanning speed for each wavelength was 0.2 s. The absorption spectra of the reference gas CH_2_F_2_ (100 ppm) in the 2360–2480 nm region and those of CH_2_F_2_ (1000 ppm) and pure H_2_O are shown in Fig. 5a,b, respectively. The pure H_2_O absorption spectra (red curve) were obtained from the high-resolution transmission molecular absorption (HITRAN) database. Compared to the absorption spectrum in Fig. 5b, the CRD absorption spectrum in Fig. 5a shows several absorption peaks other than those of CH_2_F_2_. These absorption peaks are in excellent agreement with the H_2_O absorption peaks shown in Fig. 5b. This result indicates that the high sensitivity and broad-wavelength scanning features of the CRDS allowed the detection of trace H_2_O included as an impurity in the CH_2_F_2_ reference gas. In the future, our WS-CRDS is also expected to detect HFOs (CH_2_=CFCH_3_ and CHF=CHCF_3_) that show absorption in the same wavelength region^12^.

## Conclusions

We demonstrated trace CH_2_F_2_ detection using WS-CRDS in a broad mid-IR region. WS-CRDS was performed using an ET-Cr:ZnSe laser with high wavelength controllability and single-peak nanosecond pulse operation, developed in this study. A CH_2_F_2_ detection limit of 0.66 ppm was realized along with real-time monitoring of the changes in CH_2_F_2_ concentration, thereby establishing the suitability of the proposed WS-CRDS method for leakage monitoring of CH_2_F_2_. Furthermore, the broad wavelength-tuning range of the ET-Cr:ZnSe laser allowed the detection of multiple and high-molecular-weight components that exhibit broad mid-IR absorption spectra. In the analysis of human respiration and gas released from plants, high-molecular-weight VOCs (ppb level) are often measured. Therefore, by improving the detection sensitivity, the WE-CRDS is also expected to be applied for their analysis.

## Methods

### Cr:ZnSe

A polycrystalline Cr:ZnSe (IPG Photonics, Inc.) was used as the laser medium. The concentration of Cr^2+^ was approximately 9.0 × 10^18^ cm^−3^, which was doped using the diffusion doping method^27,28^. The Cr:ZnSe was directly mounted on a copper stage without forced cooling.

### Electronic wavelength tuning via AOTF

Electronic wavelength tuning was realized using the AOTF, which consisted of TeO_2_ and a transducer, inside the laser cavity^29,30^. TeO_2_ was anti-reflection-coated for 2.0–2.7 μm. An RF signal was fed to TeO_2_ through the transducer, causing an acoustic wave to propagate in the TeO_2_, and the selected wavelength was diffracted by the acousto-optic effect. A diffraction efficiency > 90% was achieved for 2.0–2.7 μm. Here, the Cr:ZnSe laser cavity was placed along the axis of the diffracted beam. Therefore, by varying the RF signal fed to the AOTF, the lasing wavelength can be changed automatically. In our laser system, the RF was varied from 35 to 46 MHz and the RF power was set to 5 W.

## Data Availability

The data associated with this research are available from the corresponding author upon reasonable request.
